# Improved Anchorage of Ti6Al4V Orthopaedic Bone Implants through Oligonucleotide Mediated Immobilization of BMP-2 in Osteoporotic Rats

**DOI:** 10.1371/journal.pone.0086151

**Published:** 2014-01-21

**Authors:** Julia V. Wölfle, Jörg Fiedler, Lutz Dürselen, Judith Reichert, Dieter Scharnweber, Anne Förster, Bernd Schwenzer, Heiko Reichel, Anita Ignatius, Rolf E. Brenner

**Affiliations:** 1 Department of Orthopaedic Surgery, Centre of Musculoskeletal Research, University of Ulm, Ulm, Germany; 2 Division for Biochemistry of Joint and Connective Tissue Diseases, Department of Orthopaedic Surgery, Centre of Musculoskeletal Research, University of Ulm, Ulm, Germany; 3 Institute of Orthopaedic Research and Biomechanics, Centre of Musculoskeletal Research, University of Ulm, Ulm, Germany; 4 Max Bergmann Center of Biomaterials, TU Dresden, Dresden, Germany; 5 Institute of Biochemistry, TU Dresden, Dresden, Germany; Faculdade de Medicina Dentária, Universidade do Porto, Portugal

## Abstract

The aim of the present study was to test the biocompatibility and functionality of orthopaedic bone implants with immobilized oligonucleotides serving as anchor stands for rhBMP-2 and rhVEGF-A conjugated with complementary oligonucleotides in an osteoporotic rat model. Al_2_O_3_-blasted acid etched Ti6Al4V implants, carrying oligonucleotide anchor strands and hybridized with rhBMP-2 or rhVEGF-A through complementary 31-mer oligonucleotide stands were inserted into the proximal tibia of ovariectomized rats. At the time of surgery (15 weeks after ovariectomy) microCT analysis showed significantly lower bone mineral density compared to non-ovariectomized animals. Bone-implant contact (BIC) and pullout-force were not negatively affected by non-hybridized anchor strands. Twelve weeks after surgery, a significantly higher pullout force was found for BMP-2 hybridized to the anchor strands compared to non-hybridized anchor strands or native samples, and on histomorphometric analysis BIC was highest in the BMP group. Thus, we could show the biocompatibility and in vivo functionality of this modular, self-organizing system for immobilization and subsequent release of BMP-2 in vivo.

## Introduction

The coincidence of osteoarthritis and osteoporosis represents a major challenge to total joint replacement in orthopaedic surgery: Mäkkinen et al. found 74% osteopenic or osteoporotic patients in a subgroup of 53 female patients with advanced primary hip osteoarthritis scheduled for cement less total hip arthroplasty [1]. Whereas the relationship between osteoporosis and failure rate of dental implants is controversially discussed in literature [2]–[8], studies on the impact of osteoporosis on osseointegration of orthopaedic implants are less numerous and much less controversial. In an osteoporotic animal model impaired implant osseointegration into the tibia or femur has been repeatedly seen [6], [9], most recently by Stadlinger et al. [10]. Alm et al. [11] found bone loss in Gruen zone 7 after cementless total hip arthroplasty in female patients with low bone mineral density, and Aro et al. [12] recently reported that low bone mineral density negatively affects initial stability and delays stem osseointegration after cementless hip joint replacement in women. Orthopaedic implant anchorage primarily depends on osteoconduction from periimplant bone tissue. Therefore, roughness and physico-chemical properties of implant surfaces have been optimized to increase osteoconductivity [13]. A further approach to improve osseointegration of titanium implants in osteoporotic bone is surface modification with bioactive molecules encouraging bone formation. In this context the method of binding and/or release of such factors is a crucial issue [13]. Promising results have been published by Michael et al. who achieved immobilization of bioactive molecules on the implant surface in a two-step procedure: First, short strands of nucleic acids – “anchor strands” – were entrapped into a titanium oxide layer on the implant surface [14], [15]. Then, conjugates of bioactive molecules and complementary strands were hybridized to the surface modified with anchor strands [14], [16]. It has been shown that bioactive molecules thus fixed on a titanium surface retain their bioactivity: Conjugates of RGD peptides and complementary strands hybridized to anodic immobilized anchor strands increased osteoblast attachment on the titanium surface compared to the control group [16].

Promising bioactive molecules for surface modification are bone morphogenetic protein 2 (BMP-2) and vascular endothelial growth factor A (VEGF-A): BMP-2 is probably one of the most important growth factors in bone formation which is currently used in a wide range of tissue-engineering products that allow complete regeneration of bone e.g. in long bone defects or pseudarthrosis [17]–[19]. VEGF-A induces angiogenesis, which is necessary for blood supply and may also provide osteoprogenitor cells derived from multipotent pericytes for implant integration [20]. Moreover, it exerts a chemoattractive effect on human mesenchymal progenitor cells [21]–[24]. Previous in vitro studies using the oligonucleotide-mediated immobilization technology indicated that BMP-2 and VEGF-A could be effectively bound to titanium surfaces. The growth factors were released over an extended time span and induced stimulatory effects on proliferation and osteogenic differentiation of mesenchymal stem cells as well as proliferative effects on endothelial cells in vitro [25], [26]. Biological activity of released oligonucleotide conjugated BMP-2 and VEGF-A was preserved as indicated by the induction of alkaline phosphatase in C2C12 cells or van Willebrand Factor in mesenchymal stem cells in vitro [25], [26].

Since in vivo responses and functionalities have not been tested so far, the aims of this study were (i) to investigate the biocompatibility of the above-mentioned surface modification and (ii) to explore the effects of BMP-2 and VEGF-A functionalization on orthopaedic implant osseointegration in the osteoporotic bone. Therefore, Al_2_O_3_-blasted acid etched titanium (AAT) implants, with anchor strands (ODN), and with rhBMP-2 modified with 31-mer Oligonucleotides (BMP-ODN) and rhVEGF-A modified with 31-mer Oligonucleotides (VEGF-ODN) hybridized to the anchor strands were inserted into the tibia metaphysis of female rats 15 weeks after ovariectomy.

## Materials and Methods

### Ethics Statement

The animal trial was conducted according to relevant national and international guidelines; the study was approved by the local governmental authorities (Regierungspräsidium Tübingen, registration number 982). All surgery was performed under adequate anaesthesia and analgesia, and all efforts were made to minimize suffering.

### Animals

Sixty-four female ovariectomized WISTAR rats were supplied by Charles River Laboratories (Kißlegg, Germany). Ovariectomy had been performed at the age of 10 weeks. They were kept under climate-controlled conditions (21 +/− 1.5°C, 47.5 +/− 7.5% humidity, light-dark-cycle 12 hours/12 hours). Access to phytoestrogen-free diet and tap water was ad libitum. Mean body weight at the time of surgery was 397±32.2 grams.

### Preparation of the orthopaedic bone implants

**Figure 1:**
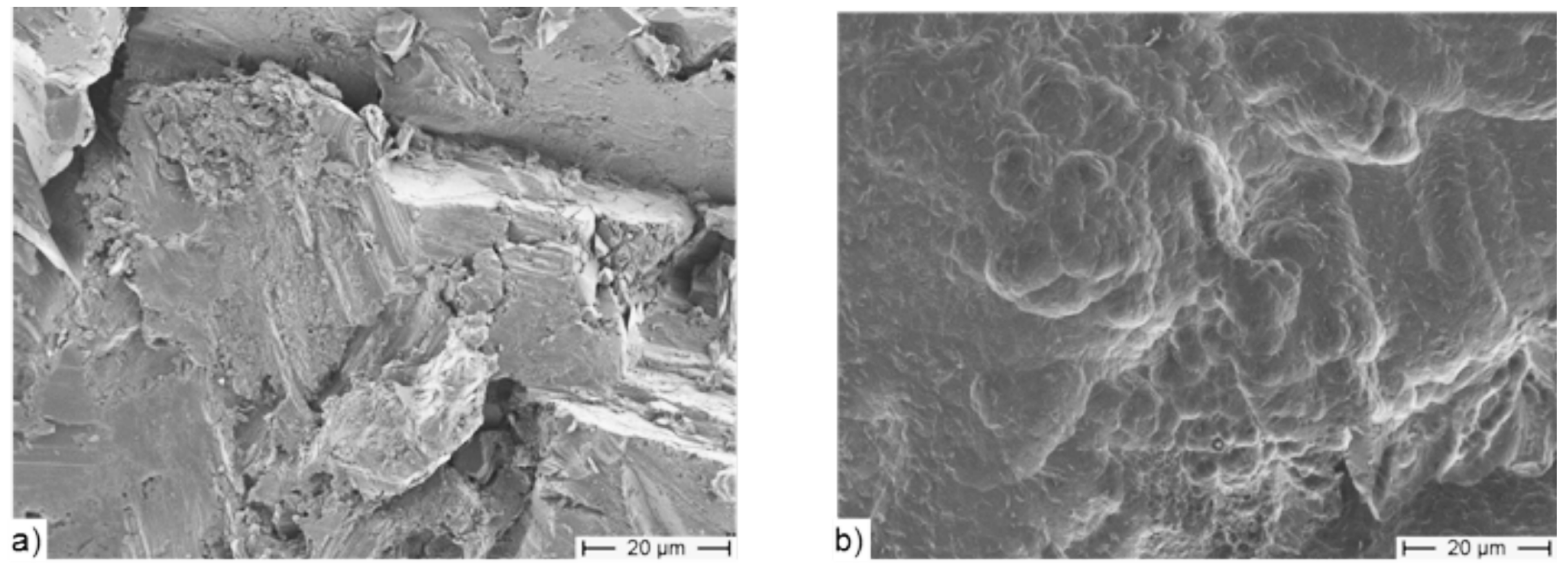
SEM-pictures of the orthopaedic implant surfaces a) before and b) after acid etching. Remaining Al_2_O_3_ particles were completely removed by etch process. In addition a gain in sub-µm structures was achieved.

The orthopaedic implants consisted of an aluminium-oxide-blasted Ti6AlV4 cylindrical rod 5.2 mm in length with a diameter of 1.6 mm (kindly provided by Peter Brehm GmbH, Weisendorf, Germany). One end of the rod was threaded (length 1.2 mm) to enable biomechanical pullout testing. The samples were acid etched for 120 s in a mixture of 0.4 M HF and 1 M HNO_3_ at room temperature and following cleaned two times 15 min in ultrapure water in a ultrasonic bath. Surface structure of the implants before and after acid etching is shown in Figure 1. Briefly, anodic polarization of the implants was performed in a custom electrochemical cell from acrylic glass with a gold wire acting as counter electrode and an Ag/AgCl reference electrode connected to the cell via a buffer-agarose salt bridge. Polarization was performed with the electrochemical system Voltalab 4.0 combined with a high-voltage booster HVB100 (Radiometer Analytical, Copenhagen). Ethanolic acetate buffer from 0.5 M acetate containing 5 M ethanol, pH 4.0 and phosphorylated 60mer single stranded oligonucleotides – briefly called anchor strand (AS) - at a concentration of 400 nM was used as electrolyte. Galvanostatic polarization at 7 mA/cm^2^ was performed until a potential of 14.5 VAg/AgCl was reached. After several desorption steps (three times in the ethanolic acetate buffer, twice in sterilized ultrapure water; each for 30 s) to remove merely adsorbed and not entrapped anchor strands the implants were packed under dry argon atmosphere and sterilized by gamma irradiation with a standard dose of 25 kGy [14], [16]. Conjugates of complementary strands of nucleic acid and recombinant human rhBMP-2 (Reliatech, Braunschweig, Germany) or recombinant human rhVEGF-A (VEGF165, Reliatech) respectively were hybridized to the surface modified with anchor strands directly before implantation under sterile conditions [25], [26]. A final washing step was performed to eliminate unbound growth factors. Calculated from the density of single stranded oligonucleotides about 2 ng rhBMP2/mm^2^ or 2 ng rhVEGF/mm^2^ were immobilized on the orthopaedic implant surface.

### Experimental design

The animals were randomly assigned 16 animals each to one of the following groups: (1) control group, (2) anchor strand group, (3) BMP-ODN group, or (4) VEGF-ODN group. According to which group they belonged to one of the following orthopaedic implants was inserted: (1) aluminium-oxide-blasted acid etched Ti6Al4V (AAT), (2) AAT with anchor strands (ODN), with (3) hybridized rhBMP-2 - modified with 31-mer Oligonucleotides (BMP-ODN) or (4) hybridized rhVEGF - modified with 31-mer Oligonucleotides (VEGF-ODN). Eight animals of each group were sacrificed after 4 weeks; the remaining animals 12 weeks after implant insertion. The left tibia was prepared for biomechanical testing, the right tibia for histomorphometric analysis, and the fourth vertebral body of the lumbar spine for analysis of bone mineral density.

To confirm reduced bone mineral density of the ovariectomized rat, four additional animals were sacrificed fifteen weeks after ovariectomy – i.e. at the time of surgery – and the bone mineral density of their fourth lumbar vertebral body was compared to the one of six otherwise identical animals who had not undergone ovariectomy.

### Surgical Procedure

Fifteen weeks after ovariectomy the above-mentioned implants were inserted into the proximal tibia on both sides. Anaesthesia was administered by means of an inhalation device (Isoflurane 2%) and subcutaneous injection of analgesics (Tramadolor 20 mg/kg). The surgical technique was similar to the one described by Dayer et al. [27]: A 10 mm incision was made at the medial aspect of the proximal tibia, and the periosteum was incised ventrally to the medial collateral ligament. A 1.7 mm drill hole was made level with the insertion of the patella tendon just ventrally to the medial collateral ligament using hand-held drills held strictly perpendicular to the longitudinal axis of the tibia. The orthopaedic implant was then inserted into the bone. The threaded part remained outside and was covered by a 2 mm tube cut off from a venous catheter (fluorinated ethylene propylene, Vasofixx Braunüle® 18 G, Braun B., Melsungen, Germany) in order to prevent osseous overgrowth. Postoperative analgesia was ensured by adding Tramadolor to the drinking water (25 milligram per litre). Antibiotics (Clindamycine 45 mg/kg) were administered subcutaneously daily on the first three postoperative days.

### Biomechanical Testing

**Figure 2:**
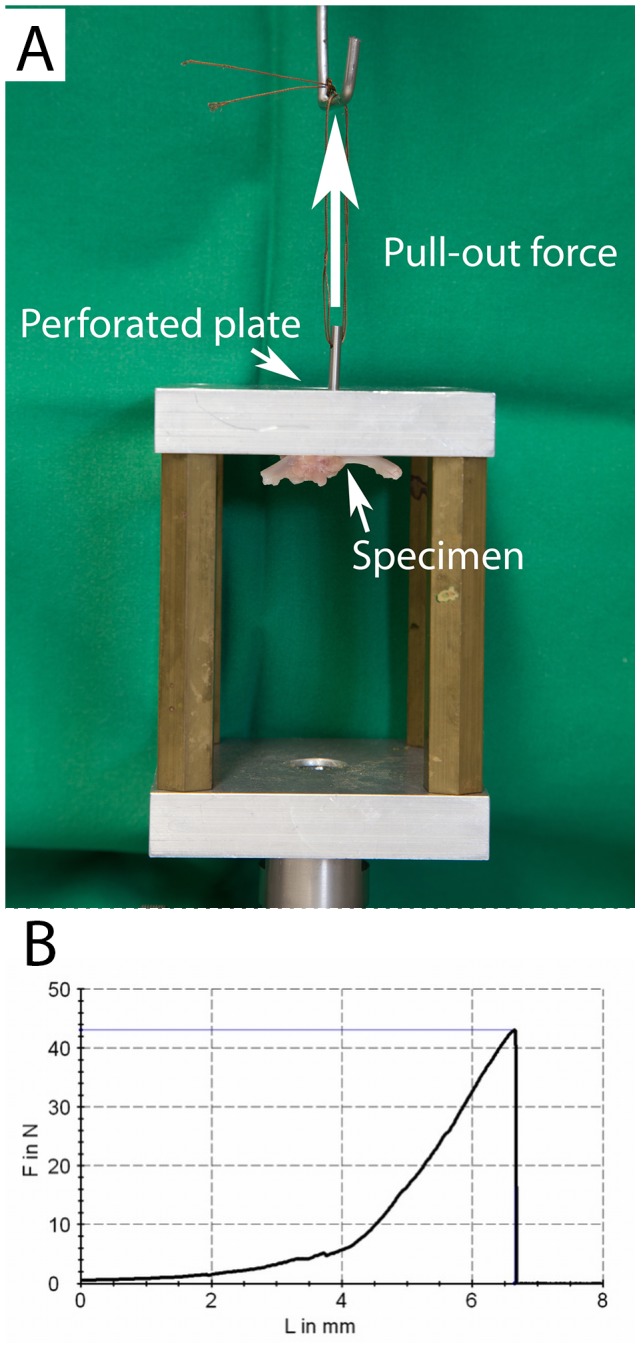
The threaded part of the orthopaedic implant was attached to a 200(HBM, Darmstadt) of a standard testing machine (Zwick, Ulm, Figure 2A). A force-displacement diagram was recorded (Figure 2B), the load occurring before the first sudden drop of the tensile force was defined as maximum pullout force. F  =  force, L  =  length/displacement.

For biomechanical testing a specifically designed cylindrical device with a matching internal thread was screwed onto the threaded part of the orthopaedic implant of the left tibia. To determine the pullout force of the implant the device was attached to a 200 N load cell (HBM, Darmstadt) of a standard testing machine (Z010, Zwick, Ulm, Germany; see Figure 2A). A force-displacement diagram (test speed 10 mm/s, preload 0.5 N) was recorded by the testing software (testXpert II, Zwick, Ulm, Germany) and the load occurring before the first sudden drop of the tensile force was defined as maximum pullout force (see Figure 2B).

### Histomorphometric measurement

The right tibia including the inserted orthopaedic implant was embedded in Technovit VLC7200 (Kulzer, Germany) and ground down to sections of 100 µm along the longitudinal axis of the tibia. Masson-Goldner staining of the sections was used to visualize connective tissue surrounding the implant. The sections were inspected and scanned with a fully automated inverted light microscope (Leica DMI6000B, Wetzlar, Germany). To quantify the amount of bone surrounding the implant the following parameters were determined semi-automatically with the aid of an imaging analysis software (MetaMorph ®, Leica, Wetzlar, Germany): The bone-to-implant contact rate was calculated by dividing the total length of bone-to-implant contact by the total length around the orthopaedic implant within the tibia. The bone density within the medullar cavity was defined as the percentage of the area of osseous tissue within a 1 mm strip of medullar cavity just distal to the orthopaedic implant.

### MicroCT scanning

The fourth vertebral body of the lumbar spine as well as two densitometric phantoms (250 mg/cm^3^ and 750 mg/cm^3^ hydroxyapatite bone-equivalent density) were scanned with a microCT system (Skyscan 1172, Kontich, Belgium) with a resolution of 10 µm. As region of interest the trabecular bone starting ten slides below the upper endplate and ending ten slides above the lower endplate was then manually selected. After calibrating the image processing software (CT Analyser V1.11.4.2, Skyscan 1172, Kontich, Belgium) with the assistance of the two-densitometric phantoms the bone mineral density of the region of interest was determined. Then, the graylevel image was reduced to a binary image using Otsu's method of histogram shape-based image thresholding. Five bone structural parameters (bone volume fraction BV/TV, connectivity density Conn.D (measuring the degree of connectivity of trabeculae normalized by TV as explained in Bouxsein et al. [28]), trabecular number Tb.N, trabecular thickness Tb.Th, and trabecular separation Tb.Sp) were then automatically determined from the region of interest (CT Analyser V1.11.4.2, Skyscan 1172, Kontich, Belgium).

### Statistical analysis

For statistical analysis the Statistical Package for Social Sciences (SPSS® Inc., IBM, version 19) was used. Due to the low number of animals per group non-parametric tests were used for statistical analysis. Mann-Whitney-U-Test was used to compare two groups (e.g. bone mineral density), Kruskal-Wallis-Test for more than two groups (e.g. pullout force). A probability value of less than 0.05 was considered to indicate statistical significance. Boxplots and median values were used to characterize the distribution of continuous variables.

## Results

### MicroCT scanning

MicroCT analysis of the four additional animals sacrificed 15 weeks after ovariectomy – i.e. at the time of the surgery – showed significantly lower bone mineral density when compared to six animals who had not undergone ovariectomy. More specific data is depicted in the supplement (Table S1).

### Histomorphometric measurement

**Figure 3:**
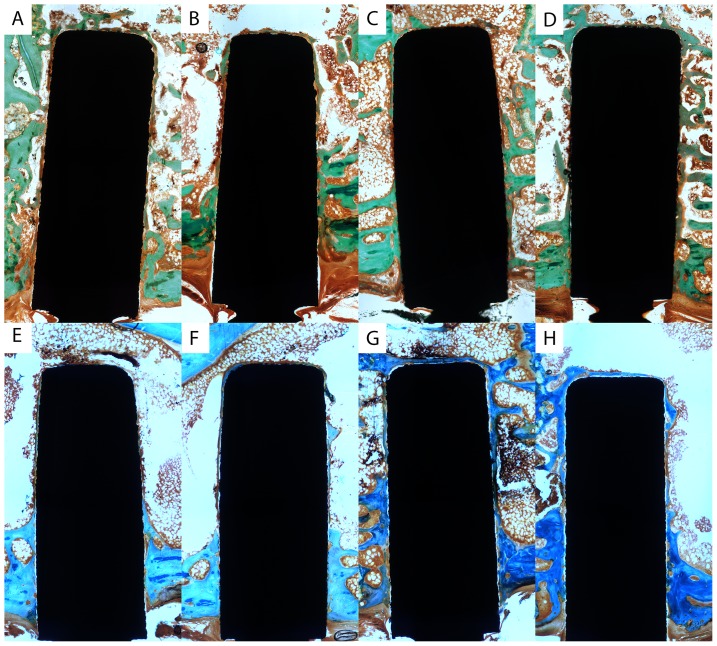
Photograph of trichrome-stains of 100 µm sections of the orthopaedic implant and surrounding osseous tissue (bone tissue in green/blue). Four weeks after surgery no relevant difference of bone-to-implant contact rate or bone density around implant is seen between AAT (A), ODN (B), BMP-ODN (C), and VEGF-ODN group (D). Twelve weeks after surgery bone-to-implant contact rate was increased within the BMP-ODN group (G) when compared to AAT (E) whereas the other groups (AAT (E), ODN (F), VEGF-ODN group (H)) showed no relevant differences. AAT: Al_2_O_3_-blasted acid etched titanium; ODN: anchor strands of oligonucleotides bound to titanium; BMP-ODN: rhBMP modified with 31-mer Oligonucleotides; VEGF-ODN: rhVEGF modified with 31-mer Oligonucleotides.

**Figure 4:**
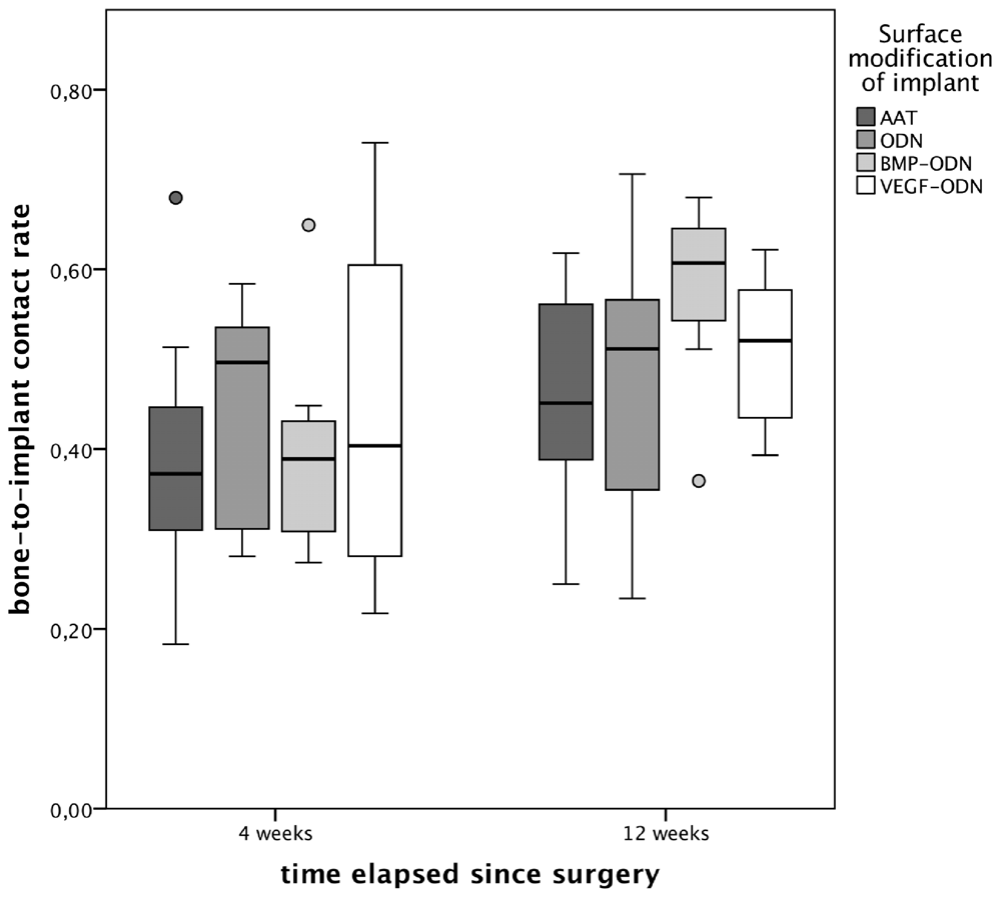
Bone-to-implant contact rate of animals sacrificed four and twelve weeks after implant insertion according to the surface modification of the orthopaedic implant. Bone-to-implant contact rate was highest in the BMP group (median 60.7%) 12 weeks after surgery though no significant difference could be found. AAT: Al_2_O_3_-blasted acid etched titanium; ODN: anchor strands of oligonucleotides bound to titanium; BMP-ODN: rhBMP modified with 31-mer Oligonucleotides; VEGF-ODN: rhVEGF modified with 31-mer Oligonucleotides.

Within the sections of the orthopaedic bone implant inserted into the tibia a thin layer of osseous tissue covered large parts of the implant. The remaining surface of the orthopaedic implant was covered to a certain extent with a thin layer of non-mineralized connective tissue. This is exemplarily shown in Figure 3 by Masson-Goldner-Stains of non-decalcified 100 µm sections of the orthopaedic implants and the surrounding osseous tissue. Four weeks after surgery no significant difference of the bone-to-implant contact rate (BIC) of the orthopaedic implants of all four groups was measureable (p = 0.927, see Fig. 4). Twelve weeks after surgery bone-to-implant contact was highest in the BMP group (median 60.7%) though no significant difference between the groups could be found (p = 0.927).

**Figure 5:**
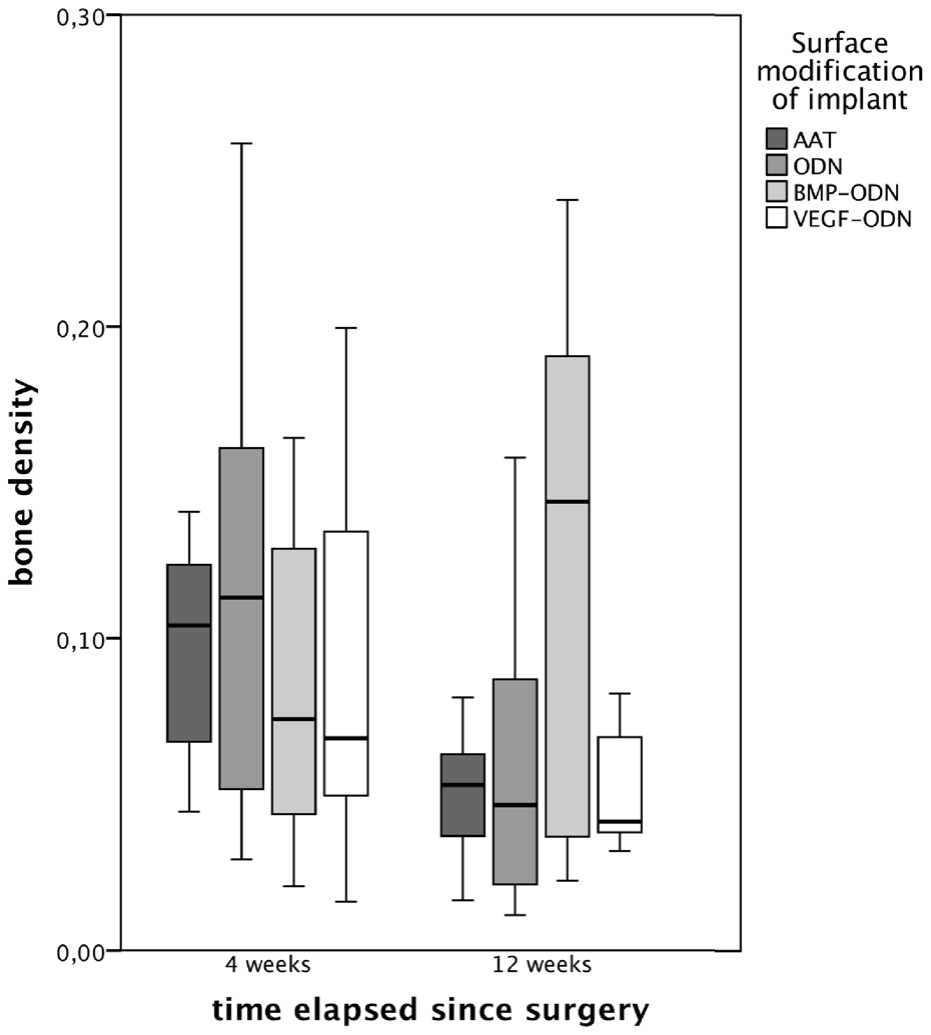
Bone density – i.e. percentage of area of osseous tissue within a 1 mm strip of medullar cavity just distal to the orthopaedic implant – of animals sacrificed four weeks and twelve weeks after implant insertion according to the surface modification of the implant. AAT: Al_2_O_3_-blasted acid etched titanium; ODN: anchor strands of oligonucleotides bound to titanium; BMP-ODN: rhBMP modified with 31-mer Oligonucleotides; VEGF-ODN: rhVEGF modified with 31-mer Oligonucleotides.

In all ovariectomized animals cancellous bone was scarcely observed within the medullar cavity. Four weeks after surgery we did not see a significant difference between the four treatment groups (p = 0.841). With exception of the BMP-ODN group we found a further decrease of the bone density in the animals sacrificed after 12 weeks when compared to the animals sacrificed after 4 weeks. As shown in Fig. 5, 12 weeks after surgery bone density was highest in the BMP-ODN group (median BD 14.3%) though we did not find a significant difference between the four groups (p = 0.488).

### Biomechanical testing

**Figure 6:**
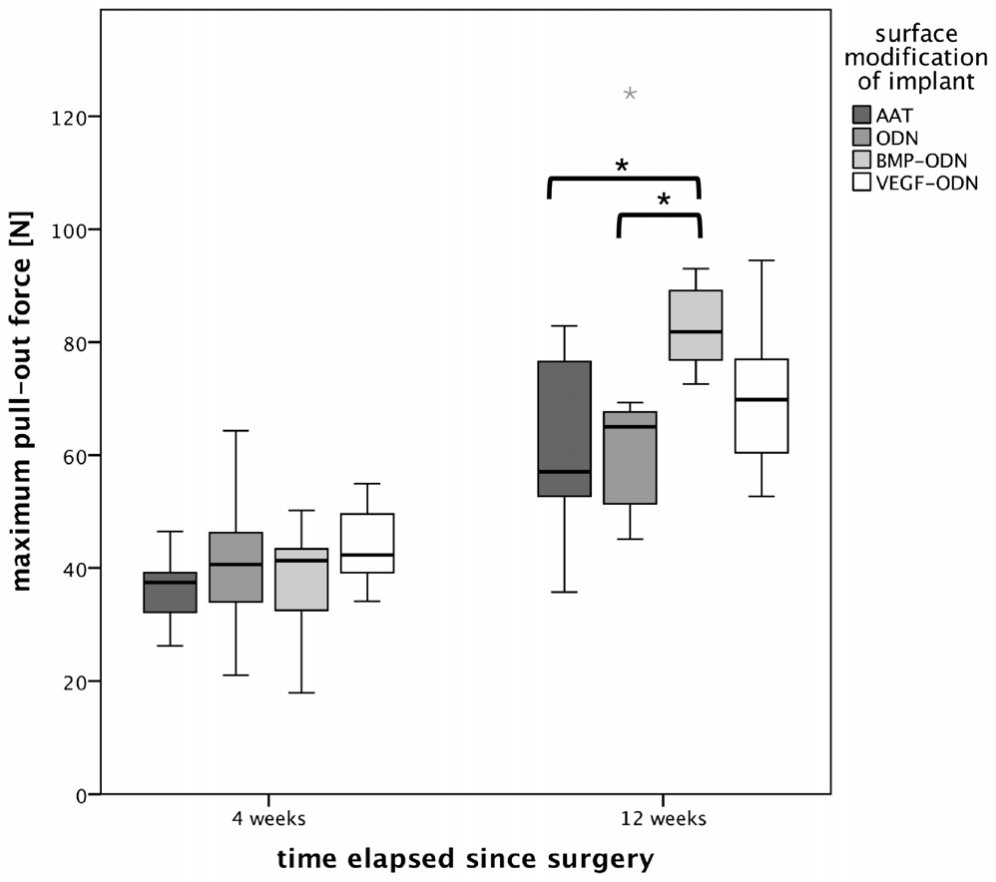
Boxplot of maximum pullout force according to surface modification four and 12 weeks after orthopaedic implant insertion. Asterisks represent significantly higher maximum pullout force in the BMP-ODN group 12 weeks after surgery (median 81.8 N) in comparison with both the AAT group (median 57.0 N) as well as the group (median 65.0 N) with p<0.05. AAT: Al_2_O_3_-blasted acid etched titanium; ODN: anchor strands of oligonucleotides bound to titanium; BMP-ODN: rhBMP modified with 31-mer Oligonucleotides; VEGF-ODN: rhVEGF modified with 31-mer Oligonucleotides.

In the animals sacrificed after four weeks maximum pullout force was not significantly different between the treatment groups. As presented in Figure 6, we found a significant difference (p = 0.034) 12 weeks after surgery. Pullout force was significantly higher in the BMP-ODN group (median 81.8 N) in comparison with both the AAT group (median 57.0 N) as well as the ODN group (median 65.0 N).

## Discussion

This animal study represents the first *in vivo* investigation of a new kind of orthopaedic implant surface functionalization, based on bioactive molecules immobilized through short complementary oligonucleotide anchor strands on a titanium surface using the specificity of nucleic acid hybridisation.

For reasons of clinical relevance ovariectomized rats were chosen for the study to simulate the compromised bone stock found in osteoporotic bone. The ovariectomized rat has been used as a model for osteoporosis in numerous studies [29]-[33]. It has been shown that after ovariectomy a biphasic pattern of cancellous bone loss occurs: During the first 100 days there is a rapid bone loss similar to high-turnover osteoporosis, which characterizes postmenopausal osteoporosis. Afterwards there is a relative stabilization at an osteopenic level with a slow bone loss resembling low-turnover osteoporosis, which is characteristic for senile osteoporosis [32], [34]. Okamura et al. found that this difference of bone turnover in the ovariectomized rat affects the bone formation around a titanium implant: There was a significantly lower bone contact rate of the implant in the animals with low bone turnover (i. e. 15 weeks after ovariectomy) when compared to the high bone turnover group (i. e. 3 weeks after ovariectomy) [31]. Since we were focusing on senile osteoporosis, we performed the orthopaedic implant insertion 15 weeks after ovariectomy, i. e. in the phase of low-turnover osteoporosis. The decrease in bone density of the ovariectomized rats was confirmed by local histomorphometric and lumbar microCT analysis. This is in agreement with previous data from ovariectomized rats indicating the development of osteoporosis in both the spine and proximal tibia in the respective age and time span after ovariectomy [35].

Our results confirm the biocompatibility of the novel surface modification: no inflammatory response surrounding the orthopaedic titanium implants inserted into the tibia regardless of their surface modification was found. The thin layer of non-mineralized connective tissue partly covering the implant surface seen in our study was much thinner than the fibrous capsule consisting of up to 30 fibroblasts, which had been described by *van den* Beucken et al. [36] when examining the biocompatibility of transponders with multilayered DNA-coatings inserted subcutaneously in rats. The varying thickness of the connective tissue found in both our study and *van den* Beucken *et al.* corresponds to the amount of nucleic acid used in both cases (5 double layers of polyanionic DNA ±300 bp/molecule vs. a single layer of 60mer ODN). With respect to biocompatibility our findings correspond with their findings because they neither described any inflammatory cells surrounding the implant or differences of the tissue response between the coated and uncoated implants [36]. Moreover, we did not observe any case of infection indicating that the sterilization by gamma irradiation before the hybridisation step was effective.

Additionally, our data suggest that BMP-2 immobilized on the orthopaedic implant surface by hybridisation to short strands of nucleic acids preserved its bioactivity in vivo and exerted a biologic effect on the surrounding tissue. This corresponds to the recently published in vitro study by Schliephake et al. [26]. They found a retarded release of rhBMP-2 using this system of nanoanchoring compared to a simple adsorption based strategy. In the respective study only 20% of the hybridized conjugated BMP-2 had been released within 4 weeks and the released conjugated BMP-2 was biologically active as indicated by similar stimulation of alkaline phosphatase in C2C12 cells compared to native BMP-2 [26]. If the release kinetic was similar under in vivo conditions a sufficient amount of bioactive BMP-2 would have been locally available in the time span after surgery during which the benefit for orthopaedic implant anchorage was observed. This in vivo response also is concordant with previous in vitro results on stimulatory effects of titanium surfaces carrying identically immobilized BMP-2 on proliferation and osteogenic differentiation of human bone marrow stroma cells [26]. Accordingly, in the animals of the BMP-ODN group sacrificed after 12 weeks we found a significantly higher pullout force when compared to both the AAT and ODN group. Similarly, the median bone-to-implant contact rate and median periimplant bone density were highest in the BMP-ODN group 12 weeks after surgery. A number of previous animal studies using different species, dosages and immobilization/carrier systems have demonstrated positive effects of BMP-2 functionalization on primary stabilization of titanium implants [37]. In these approaches BMP-2 is released early after implantation and non-physiologically high concentrations have been used [38]. With the oligonucleotide mediated immobilization strategy we could observe positive effects on orthopaedic implant anchorage with rather low concentrations of BMP-2 after the primary phase of osseointegration. This may be explained by a favourable balance of growth factor bound on the surface and the amounts of BMP-2 available in diffusible form for an extended time span. To our knowledge, comparable studies in estrogen-deficient animal models of osteoporosis do not exist so far. In a rat bone defect model of the femur, however, a positive effect of BMP-2 loaded collagen carrier has previously been described which is consistent with our results [39]. Moreover, for statin coating of titanium implants with chitosan as a carrier positive effects on bone implant contact after 2 weeks in ovariectomized rats have recently been reported [10]. Statin induces the expression of BMP-2 in osteoblastic cells and also has positive early effects on osseointegration of implants in the non-compromised situation [40].

Since a reduction of local blood supply and VEGF-expression has been previously reported in ovariectomized mice [38] positive results of the VEGF-hybridisation could be expected at the start of our study. Nevertheless, we did not observe significant effects on both bone-implant contact and pullout force up to 12 weeks after implantation although an extended in vitro release of the growth factor and in vitro bioactivity of the released conjugated VEGF-A could previously be shown [25]. This may be partly explained by recent data of Lui et al. demonstrating that intracellular effects of VEGF-A but not the extracellular interaction with membrane-bound receptors are responsible for induction of osteogenic differentiation of mesenchymal stem cells [41]. Since the outcome parameters used in our study depend on the induction of mineralized bone tissue, we might have missed associated effects on vascularization or recruitment of undifferentiated mesenchymal stromal cells. We did not observe major differences in blood vessel density around the subcutaneous implants on a macroscopic level. Therefore, we cannot exclude that the local concentrations of released conjugated VEGF-A were too low to exert a biologic response. On all things considered, the question of a possible benefit of VEGF-A for orthopaedic implant integration in estrogen-deficient osteoporotic animals clearly deserves further investigation. Published in vivo studies addressing this issue are not available so far. If an effect on vascularisation could be achieved, a combined BMP2- and VEGF-hybridisation might have the potential for further optimization of orthopaedic implant anchorage in osteoporotic bone.

## Conclusion

Our study is the first *in vivo* trial that shows the biocompatibility of an orthopaedic implant surface modification with short single strands of nucleic acids serving as anchor strands for complementary oligonucleotide conjugated bioactive molecules. Entrapped into a superficial titanium oxide layer of a Ti6Al4V implant the system is able to preserve the bioactivity of BMP-2 in vivo. Orthopaedic implant osseointegration in the compromised bone stock of estrogen-deficient osteoporotic rats was enhanced by hybridized conjugated BMP-2 molecules in a later phase of implant anchorage between 4 and 12 weeks after surgery compared with the native commercially used aluminium oxide-blasted and acid etched surface. This novel technique to functionalize biomaterial surfaces combines stable attachment of bioactive molecules and modifiable release kinetics with a high degree of flexibility in the production process. Therefore, its application for total joint replacement in osteoporotic patients seems promising.

## Supporting Information

Table S1 (DOC)

## References

[1] Makinen TJ, Alm JJ, Laine H, Svedstrom E, Aro HT (2007) The incidence of osteopenia and osteoporosis in women with hip osteoarthritis scheduled for cementless total joint replacement. Bone 40: 1041–1047. 17239668 10.1016/j.bone.2006.11.013

[2] Alsaadi G, Quirynen M, Komarek A, van Steenberghe D (2007) Impact of local and systemic factors on the incidence of oral implant failures, up to abutment connection. J Clin Periodontol 34: 610–617. 17433044 10.1111/j.1600-051X.2007.01077.x

[3] Bornstein MM, Cionca N, Mombelli A (2009) Systemic conditions and treatments as risks for implant therapy. Int J Oral Maxillofac Implants 24 Suppl:12–27. 19885432

[4] Dao TT, Anderson JD, Zarb GA (1993) Is osteoporosis a risk factor for osseointegration of dental implants? Int J Oral Maxillofac Implants 8: 137–144. 8359868

[5] Dvorak G, Arnhart C, Heuberer S, Huber CD, Watzek G, et al (2011) Peri-implantitis and late implant failures in postmenopausal women: a cross-sectional study. J Clin Periodontol 38: 950–955. 21777269 10.1111/j.1600-051X.2011.01772.x

[6] Gaetti-Jardim EC, Santiago-Junior JF, Goiato MC, Pellizer EP, Magro-Filho O, et al (2011) Dental implants in patients with osteoporosis: a clinical reality? J Craniofac Surg 22: 1111–1113. 21586959 10.1097/SCS.0b013e3182108ec9

[7] Holahan CM, Koka S, Kennel KA, Weaver AL, Assad DA, et al (2008) Effect of osteoporotic status on the survival of titanium dental implants. Int J Oral Maxillofac Implants 23: 905–910. 19014161

[8] Moy PK, Medina D, Shetty V, Aghaloo TL (2005) Dental implant failure rates and associated risk factors. Int J Oral Maxillofac Implants 20: 569–577. 16161741

[9] Yamazaki M, Shirota T, Tokugawa Y, Motohashi M, Ohno K, et al (1999) Bone reactions to titanium screw implants in ovariectomized animals. Oral Surg Oral Med Oral Pathol Oral Radiol Endod 87: 411–418. 10225622 10.1016/s1079-2104(99)70239-8

[10] Stadlinger B, Korn P, Todtmann N, Eckelt U, Range U, et al (2013) Osseointegration of biochemically modified implants in an osteoporosis rodent model. Eur Cell Mater 25: 326–340 discussion 339–340. 23832686 10.22203/ecm.v025a23

[11] Alm JJ, Makinen TJ, Lankinen P, Moritz N, Vahlberg T, et al (2009) Female patients with low systemic BMD are prone to bone loss in Gruen zone 7 after cementless total hip arthroplasty. Acta Orthop 80: 531–537. 19916684 10.3109/17453670903316801 PMC2823339

[12] Aro HT, Alm JJ, Moritz N, Makinen TJ, Lankinen P (2012) Low BMD affects initial stability and delays stem osseointegration in cementless total hip arthroplasty in women: a 2-year RSA study of 39 patients. Acta Orthop 83: 107–114. 22489886 10.3109/17453674.2012.678798 PMC3339522

[13] Schliephake H, Scharnweber D (2008) Chemical and biological functionalization of titanium for dental implants. J Mater Chem: 2404–2414.

[14] Michael J, Beutner R, Hempel U, Scharnweber D, Worch H, et al (2007) Surface modification of titanium-based alloys with bioactive molecules using electrochemically fixed nucleic acids. J Biomed Mater Res B Appl Biomater 80: 146–155. 16680695 10.1002/jbm.b.30579

[15] Beutner R, Michael J, Forster A, Schwenzer B, Scharnweber D (2009) Immobilization of oligonucleotides on titanium based materials by partial incorporation in anodic oxide layers. Biomaterials 30: 2774–2781. 19232713 10.1016/j.biomaterials.2009.01.047

[16] Michael J, Schonzart L, Israel I, Beutner R, Scharnweber D, et al (2009) Oligonucleotide-RGD peptide conjugates for surface modification of titanium implants and improvement of osteoblast adhesion. Bioconjug Chem 20: 710–718. 19368342 10.1021/bc800372e

[17] Bessa PC, Casal M, Reis RL (2008) Bone morphogenetic proteins in tissue engineering: the road from laboratory to clinic, part II (BMP delivery). J Tissue Eng Regen Med 2: 81–96. 18383454 10.1002/term.74

[18] Bessa PC, Casal M, Reis RL (2008) Bone morphogenetic proteins in tissue engineering: the road from the laboratory to the clinic, part I (basic concepts). J Tissue Eng Regen Med 2: 1–13. 18293427 10.1002/term.63

[19] Reddi AH (1998) Role of morphogenetic proteins in skeletal tissue engineering and regeneration. Nat Biotechnol 16: 247–252. 9528003 10.1038/nbt0398-247

[20] Modder UI, Khosla S (2008) Skeletal stem/osteoprogenitor cells: current concepts, alternate hypotheses, and relationship to the bone remodeling compartment. J Cell Biochem 103: 393–400. 17541947 10.1002/jcb.21423

[21] Fiedler J, Leucht F, Waltenberger J, Dehio C, Brenner RE (2005) VEGF-A and PlGF-1 stimulate chemotactic migration of human mesenchymal progenitor cells. Biochem Biophys Res Commun 334: 561–568. 16005848 10.1016/j.bbrc.2005.06.116

[22] Deckers MM, Karperien M, van der Bent C, Yamashita T, Papapoulos SE, et al (2000) Expression of vascular endothelial growth factors and their receptors during osteoblast differentiation. Endocrinology 141: 1667–1674. 10803575 10.1210/endo.141.5.7458

[23] Geiger F, Bertram H, Berger I, Lorenz H, Wall O, et al (2005) Vascular endothelial growth factor gene-activated matrix (VEGF165-GAM) enhances osteogenesis and angiogenesis in large segmental bone defects. J Bone Miner Res 20: 2028–2035. 16234976 10.1359/JBMR.050701

[24] Mayr-Wohlfart U, Waltenberger J, Hausser H, Kessler S, Gunther KP, et al (2002) Vascular endothelial growth factor stimulates chemotactic migration of primary human osteoblasts. Bone 30: 472–477. 11882460 10.1016/s8756-3282(01)00690-1

[25] Schliephake H, Strecker N, Forster A, Schwenzer B, Reichert J, et al (2012) Angiogenic functionalisation of titanium surfaces using nano-anchored VEGF - an in vitro study. Eur Cell Mater 23: 161–169 discussion 169. 22415802 10.22203/ecm.v023a12

[26] Schliephake H, Botel C, Forster A, Schwenzer B, Reichert J, et al (2012) Effect of oligonucleotide mediated immobilization of bone morphogenic proteins on titanium surfaces. Biomaterials 33: 1315–1322. 22082620 10.1016/j.biomaterials.2011.10.027

[27] Dayer R, Rizzoli R, Kaelin A, Ammann P (2006) Low protein intake is associated with impaired titanium implant osseointegration. J Bone Miner Res 21: 258–264. 16418781 10.1359/JBMR.051009

[28] Bouxsein ML, Boyd SK, Christiansen BA, Guldberg RE, Jepsen KJ, et al (2010) Guidelines for assessment of bone microstructure in rodents using micro-computed tomography. J Bone Miner Res 25: 1468–1486. 20533309 10.1002/jbmr.141

[29] Kalu DN (1991) The ovariectomized rat model of postmenopausal bone loss. Bone Miner 15: 175–191. 1773131 10.1016/0169-6009(91)90124-i

[30] Kalu DN, Liu CC, Hardin RR, Hollis BW (1989) The aged rat model of ovarian hormone deficiency bone loss. Endocrinology 124: 7–16. 2909382 10.1210/endo-124-1-7

[31] Okamura A, Ayukawa Y, Iyama S, Koyano K (2004) Effect of the difference of bone turnover on peri-titanium implant osteogenesis in ovariectomized rats. J Biomed Mater Res A 70: 497–505. 15293324 10.1002/jbm.a.30110

[32] Wronski TJ, Dann LM, Scott KS, Cintron M (1989) Long-term effects of ovariectomy and aging on the rat skeleton. Calcif Tissue Int 45: 360–366. 2509027 10.1007/BF02556007

[33] Wronski TJ, Lowry PL, Walsh CC, Ignaszewski LA (1985) Skeletal alterations in ovariectomized rats. Calcif Tissue Int 37: 324–328. 3926284 10.1007/BF02554882

[34] Ozawa S, Ogawa T, Iida K, Sukotjo C, Hasegawa H, et al (2002) Ovariectomy hinders the early stage of bone-implant integration: histomorphometric, biomechanical, and molecular analyses. Bone 30: 137–143. 11792576 10.1016/s8756-3282(01)00646-9

[35] Francisco JI, Yu Y, Oliver RA, Walsh WR (2011) Relationship between age, skeletal site, and time post-ovariectomy on bone mineral and trabecular microarchitecture in rats. J Orthop Res 29: 189–196. 20722002 10.1002/jor.21217

[36] van den Beucken JJ, Walboomers XF, Vos MR, Sommerdijk NA, Nolte RJ, et al (2007) Biological responses to multilayered DNA-coatings. J Biomed Mater Res B Appl Biomater 81: 231–238. 16969822 10.1002/jbm.b.30658

[37] Thorey F, Menzel H, Lorenz C, Gross G, Hoffmann A, et al (2010) Enhancement of endoprosthesis anchoring using BMP-2. Technol Health Care 18: 217–229. 20639598 10.3233/THC-2010-0584

[38] Ding WG, Wei ZX, Liu JB (2011) Reduced local blood supply to the tibial metaphysis is associated with ovariectomy-induced osteoporosis in mice. Connect Tissue Res 52: 25–29. 20497029 10.3109/03008201003783011

[39] Sarban S, Senkoylu A, Isikan UE, Korkusuz P, Korkusuz F (2009) Can rhBMP-2 containing collagen sponges enhance bone repair in ovariectomized rats?: a preliminary study. Clin Orthop Relat Res 467: 3113–3120. 19653054 10.1007/s11999-009-1004-6 PMC2772906

[40] Moriyama Y, Ayukawa Y, Ogino Y, Atsuta I, Koyano K (2008) Topical application of statin affects bone healing around implants. Clin Oral Implants Res 19: 600–605. 18422989 10.1111/j.1600-0501.2007.01508.x

[41] Liu Y, Berendsen AD, Jia S, Lotinun S, Baron R, et al (2012) Intracellular VEGF regulates the balance between osteoblast and adipocyte differentiation. J Clin Invest 122: 3101–3113. 22886301 10.1172/JCI61209 PMC3428080

