# Supplementary material for: Improved Anchorage of Ti6Al4V Orthopaedic Bone Implants through Oligonucleotide Mediated Immobilization of BMP-2 in Osteoporotic Rats
Source: PLoS One. 2014 Jan 21;9(1):e86151. doi: 10.1371/journal.pone.0086151 (PMC3897651; doi:10.1371/journal.pone.0086151)
Supplement: Table S1 — (DOC) [file pone.0086151.s001.doc]

| **Table S1:** Micro-CT analysis of the 4^th^ vertebral body of four animals sacrificed 15 weeks after ovariectomy (OVX) compared to six animals without ovariectomy (control). | | | |
| --- | --- | --- | --- |
|  | OVX (median) | control (median) | p-value |
| BMD | 0.38 g/cm^3^ | 0.478 g/cm^3^ | p=0.033 |
| BV/TV | 27.2 % | 43.5 % | p=0.011 |
| Tb.Th | 0.10 mm | 0.10 mm | p=0.670 |
| Tb.N | 2.8 1/mm | 4.2 1/mm | p=0.011 |
| Tb.Sp | 0.293 mm | 0.188 mm | p=0.011 |
| Conn.D | 61.9 1/mm^3^ | 120.5 1/mm^3^ | p=0.019 |
| BMD bone mineral density, BV/TV bone volume fraction, Conn.D connectivity density, Tb.N trabecular number, Tb.Sp trabecular separation, Tb.Th trabecular thickness. | | | |
